# A Study on Origin Traceability of White Tea (White Peony) Based on Near-Infrared Spectroscopy and Machine Learning Algorithms

**DOI:** 10.3390/foods12030499

**Published:** 2023-01-21

**Authors:** Lingzhi Zhang, Haomin Dai, Jialin Zhang, Zhiqiang Zheng, Bo Song, Jiaya Chen, Gang Lin, Linhai Chen, Weijiang Sun, Yan Huang

**Affiliations:** 1 College of Horticulture, Fujian Agriculture and Forestry University, Fuzhou 350002, China; 2 LiuMiao White Tea Corporation, Fuding 355200, China; 3 Fujian Rongyuntong Ecological Technology Limited Company, Fuzhou 350025, China; 4 Fu’an Tea Industry Development Center, Fu’an 355000, China; 5 Institute of China White Tea, Fuding 355200, China; 6 Anxi College of Tea Science, Fujian Agriculture and Forestry University, Quanzhou 362400, China

**Keywords:** white tea, near-infrared spectroscopy, geographic origin traceability, machine learning

## Abstract

Identifying the geographical origins of white tea is of significance because the quality and price of white tea from different production areas vary largely from different growing environment and climatic conditions. In this study, we used near-infrared spectroscopy (NIRS) with white tea (*n* = 579) to produce models to discriminate these origins under different conditions. Continuous wavelet transform (CWT), min-max normalization (Minmax), multiplicative scattering correction (MSC) and standard normal variables (SNV) were used to preprocess the original spectra (OS). The approaches of principal component analysis (PCA), linear discriminant analysis (LDA) and successive projection algorithm (SPA) were used for features extraction. Subsequently, identification models of white tea from different provinces of China (DPC), different districts of Fujian Province (DDFP) and authenticity of Fuding white tea (AFWT) were established by K-nearest neighbors (KNN), random forest (RF) and support vector machine (SVM) algorithms. Among the established models, DPC-CWT-LDA-KNN, DDFP-OS-LDA-KNN and AFWT-OS-LDA-KNN have the best performances, with recognition accuracies of 88.97%, 93.88% and 97.96%, respectively; the area under curve (AUC) values were 0.85, 0.93 and 0.98, respectively. The research revealed that NIRS with machine learning algorithms can be an effective tool for the geographical origin traceability of white tea.

## 1. Introduction

Tea (*Camellia sinensis* (L.) O. Kuntze) is the second most consumed beverage in the world after water [1]. It contains rich secondary metabolites that are strongly associated with benefits to human health, such as free amino acids, polyphenols and alkaloids which are good for human health, create a complex and varied taste and attractive aroma [2,3]. In general, according to the degree of fermentation and processing techniques, tea is classified into six categories: green tea (unfermented, enzyme inactivation), white tea (slightly fermented, withering), yellow tea (partly fermented, heaping for yellowing), Oolong tea (partial-fermented, fine manipulation), black tea (fully fermented, fermentation), and dark tea (post-fermented, pile) [4,5]. Unlike other kinds of tea, white tea has the simplest producing process with only two steps: withering and drying [6]. In recent years, white tea has become increasingly popular with an ever-growing international market demand and public interest because of its unique flavor and health benefits [7]. The flavor and quality of white tea are often affected by origins, and the origin is an important basis for consumers to make a purchase. Fuding white tea is the best-known white tea in China, and it is popular among consumers as a China-Europe Geographical Indication Product with a higher commercial value compared to white tea produced in other areas. It has been reported that Fuding white tea sold on the market is far more than the actual production [8]. It is difficult for consumers to distinguish white tea from different producing areas only by its appearance, which may affect the value assessment of white tea products. Therefore, a reliable and fast method is increasingly needed to identify and trace the white tea produced in different origins, thereby providing strong technical support the high-quality development of the white tea industry.

Until now, the identification of tea origins has mainly relied on professional experts to conduct sensory evaluation by tea appearance and flavor, and the results are easily influenced by experts’ physical conditions and mental factors, leading to the results being more subjective and lacking repeatability [9]. In recent years, proton transfer reaction time of flight-mass spectrometry [10], inductively coupled plasma optical emission spectrometry and inductively coupled plasma mass spectrometry [11] have been used for the origin tracing of white tea, but these methods all have problems of being time-consuming, costly and complex to analyze, which make it difficult to promote and utilize in industrial application. Near-infrared spectroscopy (NIRS), as a green analytical technique with high efficiency, high accuracy, and convenience, has proven its applicability in the field of fuel [12], medicine [13] and wine [14], and has shown its advantages in the traceability of the origin of other teas. Jin et al. [15] used near-infrared spectral data combined with an extreme learning machine to build an origin traceability model for Taiping Houkui green tea in a narrow region, and the accuracy rate of the optimized model could reach 95.35%. Ren et al. [16] used a factorization method combined with NIRS data to establish rapid identification model of black tea growing regions, and the identification accuracy of black tea from different geographical regions was 94.3%. Yan et al. [17] used partial least squares discriminant analysis and a NIRS-established model to discriminate Anxi-Tieguanyin’s (oolong tea) authenticity, the best model’s specificity and sensitivity could reach 0.931 and 1.000.

In recent years, machine learning algorithms have also been gradually used to identify food products’ producing areas, authenticity. Xu et al. [18] successfully identified 16 kinds of millet origins based on Vis-NIR data combined with machine learning algorithms, with F-Score values up to 99.5% for random forest (RF) and support vector machine (SVM) models, and 99.1% for K-nearest neighbor (KNN) models. Zhang et al. [19] combined hyperspectral data with SVM algorithm to successfully achieve a fast and nondestructive identification of salted sea cucumbers, over-salted sea cucumbers and sugar-treated sea cucumbers, with the best model achieving 100% accuracy. Liu et al. [20] combined the hyperspectral data with the PCA algorithm and SVM algorithm to achieve a fast and nondestructive identification of green tea origins and the exact processing month, and the correct recognition rate of the best origin identification model could reach 97.5%; the correct recognition rate of the best processing-month recognition model could reach 95%. The above results demonstrate that NIRS combined with machine learning algorithms has the potential to achieve rapid and nondestructive identification of white tea’s origins, but there is no relevant report about NIRS application in identifying white tea origins.

Therefore, the main purpose of this paper is to investigate the potential and possibility of using NIRS combined with different preprocessing, feature extraction and machine learning algorithms analysis as a fast and nondestructive tool to identify and classify white tea according to geographically larger production areas (different provinces of China, DPC), narrow range of origins (different districts of Fujian Province, DDFP) and the authenticity of China-Europe Geographical Indication Product (authenticity of Fuding white tea, AFWT). This paper describes the systematic and comprehensive evaluation of the applicability of NIRS as a traceability tool for white tea.

## 2. Materials and Methods

### 2.1. Set of White Tea (White Peony) Samples and Preliminary Treatment

**Figure 1:**
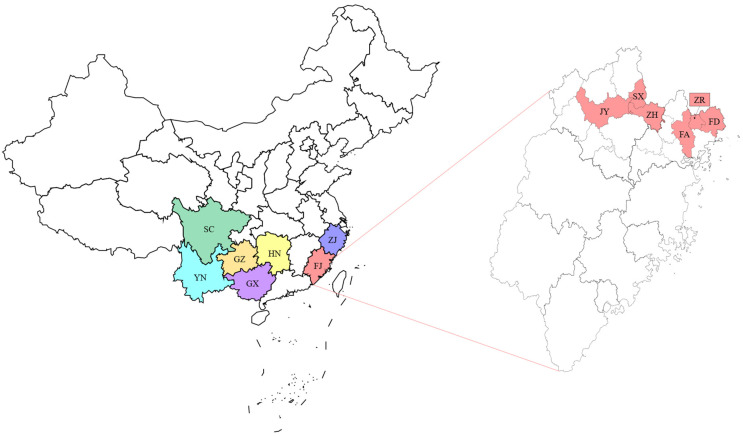
Sampling locations of white tea samples. (On the left is the geographical locations of seven different provinces in China, including FJ, GZ, HN, SC, YN, ZJ and GX; on the right is the portion zooming in to show the white tea production districts in Fujian Province, including FD, FA, ZH, SX, JY and ZR).

A sample of 579 white teas (first-grade white peony) produced in the spring of 2021 (*n* = 233) and 2022 (*n* = 346) was considered for this study. Samples were collected from local markets in seven provinces of China, including Fujian Province (FJ, *n* = 389), Guizhou Province (GZ, *n* = 55), Hunan Province (HN, *n* = 40), Sichuan Province (SC, *n* = 40), Yunnan Province (YN, *n* = 34), Zhejiang Province (ZJ, *n* = 12) and Guangxi Province (GX, *n* = 9). The 389 samples from Fujian Province were collected from six different districts: Fuding (FD, *n* = 192), Fu’an (FA, *n* = 51), Zhenghe (ZH, *n* = 49), Songxi (SX, *n* = 32), Jianyang (JY, *n* = 29) and Zherong (ZR, *n* = 36). Figure 1 shows the locations of white tea samples. White tea produced in different districts of Fujian Province has a similar quality due to its similar geographical location and could be further divided into Fuding white tea and non-Fuding (Non-FD, *n* = 197) white tea.

### 2.2. Spectra Acquisition

The samples’ NIRS was collected using an Antaris II FT-NIR spectrophotometer (Thermo Scientific, Waltham, MA, USA). The NIRS was operated at a temperature of 25 °C and humidity < 70%; workflow spectral acquisition workflow parameters were set as wave number range 4000–10,000 cm^−1^, scan interval 3.856 cm^−1^, 64 times, and resolution 8.0 cm^−1^. To ensure the reliability of the NIRS detection data, the samples were scanned once for the background before the acquisition, and the air background spectra was deducted to reduce the influence of environmental factors on the spectra data, the spectra of each sample were collected three times, and the average spectrum was taken as the original spectral data. The spectra were saved as absorbance using TQ Analyst software (Thermo Nicolet Corporation, Madison, WI, USA) for subsequent analysis.

### 2.3. Spectral Pretreatment

Due to the influence of electrical noise, light scattering and other environmental factors, it is inevitable to have baseline drift and high-frequency noise in NIRS data. To further eliminate the influence of the environmental factors on the original spectra (OS), continuous wavelet transform (CWT), minmax normalization (Minmax), standard normal variate (SNV) and multiplicative scattering correction (MSC) were used as four preprocessing algorithms in MATLAB (MATLAB R2016a, Mathworks) for spectra correction. The CWT algorithm was used to correct the baseline drift and eliminate high-frequency noise; the Minmax algorithm was chosen to strengthen the data; the SNV and MSC algorithms were used to correct the scattering and eliminate the effects caused by the inhomogeneity of tea powder particle size and the nonconstant light range [21,22,23,24]. The choice of wavelet parameters (wavelet basis and decomposition scale) in CWT was crucial and directly determined the merits of the subsequent models [25]. After trial calculation and analysis, the db4 wavelet basis of the Daubechies family was chosen in this study, and the decomposition scale was set as 64.

### 2.4. Extraction of Characteristics

A large amount of redundant information existed in the continuous wavenumbers of NIRS, which closely related to feature information. Computational speed and accuracy can be easily affected due to excessive data if we use all the data to build models. Therefore, to better reduce the computational burden of models, we applied dimensionality reduction of spectra data to characterize the vast majority of information of the spectra by extracting feature vectors or wavenumbers. In this study, principal component analysis (PCA), linear discriminant analysis (LDA), and successive projection algorithm (SPA) were used to perform data dimensionality reduction, and the above algorithms were all implemented in Python v3.8.5.

Among these methods, PCA is often applied to reduce the dimensionality of spectra in agricultural and livestock products and has been proven to be an effective spectra dimensionality reduction method, which can extract features from a large amount of data and convert them into the data set that still contains most of the valid information but has a smaller dimensionality. Thus, the original data information is retained to the greatest extent [26]. Therefore, the PCA method is optimal and the most commonly used.

LDA is a supervised feature extraction method, which is based on the principle that all sample points are projected onto a high-dimensional line, so that the projections of the sample points of the same class are as close as possible, while the projections of the sample points of different classes are distributed as scattered as possible [27].

SPA is a forward circular feature extraction method, which can extract the information of effective predictive response variables from the original spectral matrix by continuous projection, and minimize the covariance effect between the spectral variables to maximize the predictive ability of the selected response variables [28]. The wavenumber with the largest projection vector and the smallest covariance with the wavenumber in the feature set is selected into the feature set. The number of characteristic wavenumbers is determined by the root to mean square error (RMSE) of the internal complete cross-validation of the calibration set, and the number of features and characteristic wavenumbers corresponding to the minimum RMSE value are the best values [29].

### 2.5. Establishment and Evaluation of Models

Machine learning algorithms are widely used in the analysis and utilization of NIRS data, but so far, no classifier has shown its superior advantages in all cases. Hence, using multiple classifiers for modeling is better for constructing high-quality models. In this paper, we adopted three classical machine learning algorithms, including K-nearest neighbor (KNN), random forest (RF) and support vector machine (SVM), and combined the NIRS data processed by different pre-processing and feature extraction algorithms to build models and optimize the model parameters, to systematically and comprehensively explore the optimal process for the construction of white tea origin traceability models. All model constructions were based on Python v3.8.5, and the evaluation parameter tables were made with Excel. Before the models were constructed, the data were divided into four equal parts, of which three parts were used as the training set and one part was used as the validation set. The training set was used to construct the traceability model; the validation set was used to evaluate the source prediction ability of the model for new samples.

The KNN classification algorithm is one of the simplest machine learning algorithms with mature theory and wide application. Its principle is to judge the attributes based on the category of the nearest k points when predicting new values, which is simple, fast, and insensitive to outliers. The selection of k-value will have a significant impact on the results of the algorithm, when the k-value is small, the overall complexity of the model will rise and be prone to overfitting. When the k-value is large, it will make the training set instances far away from the validation set samples, which influences the prediction’s making the prediction errors occur [30]. In practice, the k-value is generally chosen as a small value, and cross-validation is subsequently used to select the optimal k-value, and the initial k-value was set as 3 in this study after in-depth analysis.

RF is a supervised integrated classification algorithm that emerged mainly to solve the problem of large errors and over-fitting that may occur in a single decision tree. RF performs well in classification problems, with great potential to become the classifier with optimal effect in each case. The model consists of many decision trees, but there is no association with each other. When judging or predicting a new sample after getting the forest, each decision tree in the forest will be judged separately to distinguish which category the sample belongs to and compare which category has the highest number of choices to make a judgment on the sample category; it is crucial to decide how many trees in this model should have [31,32]. After the trial calculation, the number of trees in this study was initially set as 20 for the subsequent comparative analysis.

In recent years, SVM has become one of the most widely used and effective machine learning algorithms for use in tea. It is an algorithm that uses a kernel function to map the input n-dimensional data to a K-dimensional feature space (K > n) to perform classification by a high-dimensional feature space. To improve the model quality, all SVM models in this paper were based on the radial basis function (RBF) kernel function, which could reduce the computational complexity of the training process and has good performance under the general smoothing assumption; at the same time, the determination of the optimal values of the penalty parameter C and gamma parameter is also crucial, and the accuracy of the SVM models depends on the combination of these two parameters [33]. The accuracy of the SVM model depends on the combination of these two parameters. After trial calculations, C = 100 and gamma = 0.1 were used as the initial modeling parameters in this study.

The model performance was preliminarily evaluated using recognition accuracy (RA) and area under curve (AUC). In detail, RA is often used to evaluate the predictive ability of the model, and its value range is between 0 and 100%. The larger the value is, the better the predictive ability of the model for new samples. AUC is often used to evaluate the generalization ability of the model. The better the generalization ability of the model, the better the ability to classify new samples correctly. The value range of AUC is from 0 to 1.0 and is positively correlated with the quality of the model [34,35]. When the preliminary evaluation parameters of models are the same, to further evaluate the discriminant and generalization ability, four-fold cross-validation is used to verify the accuracy of the model. Four-fold cross-validation refers to dividing the original data into four subsets equally, making each subset data as a validation set respectively, and the rest data as a training set to obtain four model performance parameters, and using the average of these four models RA as the performance index of this classifier [36]. The confusion matrix is often used to evaluate the classification effect of each group, reflecting the relationship between the real category of the sample data and the prediction results, and quantifying the details of classification more intuitively [37]. In this paper, the confusion matrices were used for the classification details evaluation of the best models obtained.

### 2.6. Data Analysis

The raw NIRS saved the spectrum as absorbance through TQ Analyst (Thermo Nicolet Corporation) software for subsequent analysis. MATLAB (MATLAB R2016a, Mathworks) software was used to preprocess the raw spectrum and draw all spectra. Python v3.8.5 software was used to extract features, build models and draw 3D models. The model evaluation tables and parameter optimization diagrams were generated in Excel. Confusion matrices were generated by TBtools software (Guangdong, China).

## 3. Results and Discussion

### 3.1. Spectral Analysis

**Figure 2:**
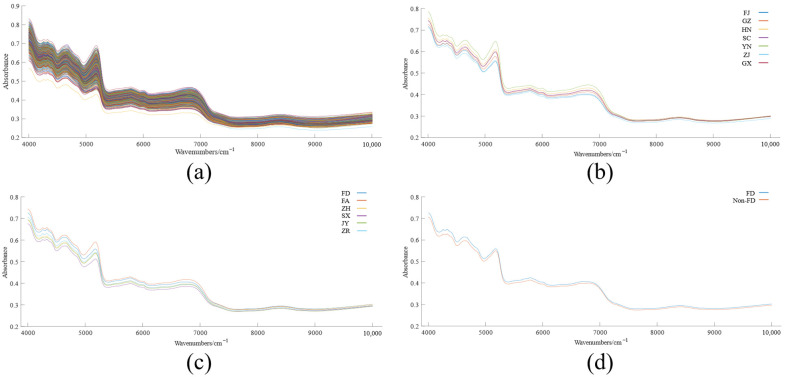
NIRS of white tea samples. (**a**) Original NIRS of 579 white tea samples; (**b**) average NIRS of white tea from different provinces; (**c**) average NIRS of white tea from different production districts in Fujian Province; (**d**) average NIRS of FD white tea and non-FD white tea.

Figure 2a shows the initial NIRS of 579 white tea samples in the 4000–10,000 cm^−1^ band. The trend of absorbance values in each band tended to be consistent without any significant differences. With the increase of wavenumber, the absorbance values showed an overall decreasing trend, and the range of variation was between 0.239 and 0.833.

**Figure 3:**
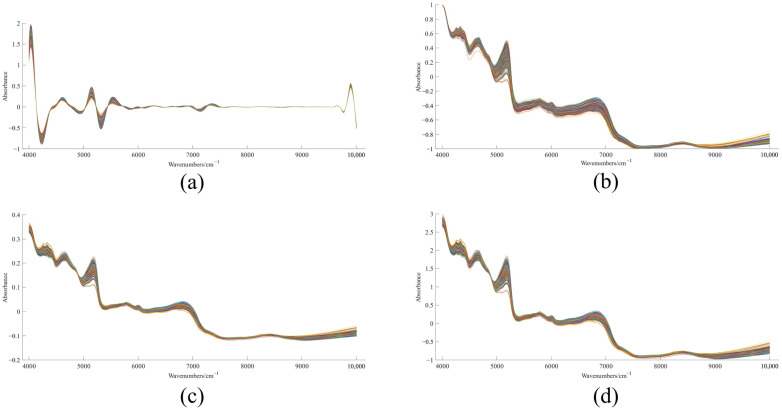
The effects of different spectral pretreatment methods. (**a**) CWT, (**b**) Minmax, (**c**) MSC, (**d**) SNV.

To visualize the differences in the NIRS of white tea from different origins, three different average spectra were plotted based on the OS data: (1) the samples were classified by DPC (FJ vs. GZ vs. HN vs. SC vs. YN vs. ZJ vs. GX, Figure 2b); (2) the samples were classified by DDFP (FD vs. FA vs. ZH vs. SX vs. JY vs. ZR, Figure 2c); (3) the samples were classified by AFWT (FD vs. Non-FD, Figure 2d). With each average spectrum observed, it could be found that the absorbance values fluctuated significantly in the range of 4000–7200 cm^−1^, and the average spectra could be largely separated from each other, indicating that the white tea samples of different geographical origins have different absorbance increases and decreases in this band, which indicates a correlation between the spectral information and the origin. The overlap among the average spectra from 7200–10,000 cm^−1^ in Figure 2b,c indicates there was less effective information related to the origin in this band; in addition, the fluctuation of the band tends to be flat without obvious peaks and valleys, which means that the characteristic information in this band was not obvious and the signal-to-noise ratio was low.

### 3.2. Spectral Pretreatment

The OS contained a large amount of chemical information about the samples, but there existed obvious problems that baseline drift and spectra peak overlap, which made it difficult to trace the geographical origin of white tea by the OS only. To further optimize the OS data, spectral preprocessing was performed with CWT, Minmax, MSC and SNV.

We can see clearly from the spectrogram changes that all four treatments led to great changes in the spectra morphology. Figure 3a applied CWT for spectral preprocessing; the degree of morphological transformation was the largest among the four preprocessing methods, baseline drift, background interference and noise phenomena were eliminated, the spectral peaks were clearer and the segments of difference information were more obvious. The minmax algorithm (Figure 3b) condensed the spectral absorbance values into −1 to 1, which augmented the data and eliminated the influence of data outline and the range of values, and the subsequent could make the constructed model converge faster and improve the model performance. To eliminate the influence of the uneven size of tea powder particles and the scattering generated, SNV with MSC was used for preprocessing (Figure 3c,d), and the scattering interference in the spectra was eliminated after processing, and the feature information was more prominent. Compared with OS, the pretreatment could effectively eliminate the signal interference caused by light scattering and baseline drift in the spectra, but the treated spectrograms still could not visually distinguish the differences among the production areas, which might be due to the more similarity in the composition and content of inclusions in white tea from different producing areas. Consistent with OS, the fluctuations at 7200–10,000 cm^−1^ of the four pre-treated spectra were still flat and the feature information was not obvious. To reduce the data’s dimensionality in the model and improve the model calculation’s speed and quality, this segment was discarded in the subsequent model construction [9].

### 3.3. Extraction of Characteristics

In this study, the NIRS data in the range of 4000–10,000 cm^−1^ was obtained; after preprocessing and comparison analysis, it was decided to use 4000–7200 cm^−1^ for the establishment of the origin traceability models of white tea. Using all data in the range to build models may negatively affect the operation speed and accuracy due to a large amount of data. Therefore, the processes of dimensionality reduction were performed to extract features with lower dimensionality to characterize the spectra data information. This study used PCA, LDA and SPA to achieve dimensionality reduction, and the optimal dimensionality reduction method was determined based on the modeling results.

#### 3.3.1. PCA

The characteristic vectors in OS and preprocessed NIR spectra by CWT, Minmax, MSC and SNV were extracted by PCA, and the results are shown in Table 1. The table shows the extracted eigenvalues and cumulative contributions of the first 15 principal components, and the number of model input principal components was screened based on the principle that the eigenvalue is >1 and the cumulative contribution is >80%.

In the NIRS data matrix of white tea origins classified by DPC, the number of feature vectors obtained by OS was 4; the number of feature vectors obtained by CWT, Minmax, MSC and SNV preprocessed spectra were 11, 7, 7 and 7, respectively; in the NIRS data matrix of white tea origins classified by DDFP or AFWT, the number of feature vectors obtained by OS was 4; the number of feature vectors obtained by CWT, Minmax, MSC and SNV preprocessed spectra were 10, 6, 7 and 7, respectively. The cumulative contribution was >80%, which was consistent with the principle, and models were subsequently constructed based on the screened principal components.

#### 3.3.2. LDA

LDA is commonly used as a classifier in the field of tea. However, the research on using LDA for NIRS feature extraction and building was rarely reported involving white tea recognition models based on the extracted feature vectors combined with classifiers.

LDA can reduce the dimension of the data matrix to the number of categories minus 1. To reduce the dimension without losing too much original information, all dimensions obtained by LDA dimension reduction would be used for subsequent modeling. Therefore, the number of feature vectors obtained using LDA for DPC, DDFP and AFWT data matrices was 6, 5 and 1, respectively.

#### 3.3.3. SPA

**Figure 4:**
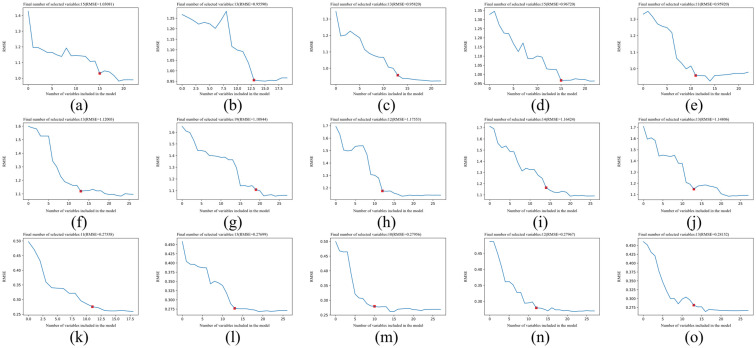
SPA treatment obtained RMSE iterative descent curve. (**a**) DPC-OS-SPA; (**b**) DPC-CWT-SPA; (**c**) DPC-Minmax-SPA; (**d**) DPC-MSC-SPA; (**e**) DPC-SNV-SPA; (**f**) DDFP-OS-SPA; (**g**) DDFP-CWT-SPA; (**h**) DDFP-Minmax-SPA; (**i**) DDFP-MSC-SPA; (**j**) DDFP-SNV-SPA; (**k**) AFWT-OS-SPA; (**l**) AFWT-CWT-SPA; (**m**) AFWT-Minmax-SPA; (**n**) AFWT-MSC-SPA; (**o**) AFWT-SNV-SPA. (―—RMSE iterative descent curve; ▉—Optimal number of variables).

Figure 4 shows the number of feature wavenumbers extracted by SPA. As can be seen from Figure 4, the RMSE reached the minimum value when a specific number of wavenumbers was selected; and after that, although the RMSE still fluctuated and decreased, the decrease was small and led to an increase in the selected number of wavenumbers, so there was no need to increase the number of dimensions to pursue a smaller RMSE. Figure 4a–e shows the iterative RMSE decline curves of the white tea NIRS data matrix of DPC obtained by SPA, from which it could be seen that the number of feature wavenumbers obtained from the final feature extraction was 15, 13, 13, 15 and 11; Figure 4f–j shows the iterative RMSE decline curves of the white tea NIRS spectra data matrix of DDFP obtained by SPA, from which it could be seen that the number of feature wavenumbers obtained from the final feature extraction was 13, 19, 12, 14 and 13; Figure 4k–o shows the iterative RMSE decline curves of the white tea spectra data matrix of AFWT obtained by SPA, from which it could be seen that the number of feature wavenumbers obtained from the final feature extraction was 11, 13, 10, 12 and 13.

### 3.4. Models Evaluation and Optimization

The KNN, RF and SVM algorithms were used to train the models on the spectra data to achieve the following objectives: (1) white tea classified by DPC (FJ vs. GZ vs. HN vs. SC vs. YN vs. ZJ vs. GX); (2) white tea classified by DDFP (FD vs. FA vs. ZH vs. SX vs. JY vs. ZR); (3) white tea classified by AFWT (FD vs. Non-FD). In this paper, the joint evaluation of RA and AUC was used to preliminarily evaluate the performance of models, and the parameters of the models with the best performance were optimized.

#### 3.4.1. Models Evaluation of White Tea’s Origins Classified by DPC

Table 2 shows the evaluation parameters of the model obtained from NIRS data combined with different preprocessing, feature extraction and machine learning algorithms for the identification of white tea from geographically larger production areas (DPC, including FJ, GZ, HN, SC, YN, ZJ and GX). The number of training set samples of all DPC recognition models was 434, and the number of validation set samples was 145. The number of modeling features before dimensionality reduction was 831, and after dimensionality reduction, the number of modeling features was reduced to about 10, which greatly reduced the computational task and improved the computing speed. The recognition accuracy ranged from 66.90 to 86.90%, and the AUC values were in the range of 0.50 to 0.83. The majority of the obtained models have RA > 70% and AUC > 0.65, which indicated that the NIRS data of the samples were highly correlated with the classification and identification of white tea production provinces, and their combination with machine learning algorithms could effectively identify white tea from different production provinces. Therefore, the research method proposed in this study was reasonable and effective for tracing white tea production provinces.

In the established DPC recognition model of white tea, the RA of KNN, RF and SVM models based on OS were 73.10%, 75.17% and 75.86%, respectively, and the AUC values were 0.62, 0.65 and 0.63, respectively. The pretreatment of the initially established KNN and RF models were significantly improved with CWT, Minmax, MSC and SNV, the RA and AUC, and the model prediction and generalization ability were further improved. Compared with the results of the DPC-OS-SVM model, only CWT and SNV algorithms achieve the purpose of optimized models.

After further combining the feature extraction algorithms, the dimensionality of the data was significantly reduced, but the performance of most models was not further improved by the feature extraction algorithms. The number of models whose model quality was further improved after dimensionality reduction of NIRS data used PCA, LDA and SPA algorithms were 11, 5 and 2, respectively. In the process of establishing geographically larger production area recognition models, the dimensionality reduction algorithm PCA performed the best, accomplished the reduction of data dimensionality for the vast majority of models, reduced the computational task and improved the model computing speed. The number of model quality improved using LDA was not as good as PCA, but it had the least number of feature dimensions after dimensionality reduction, and with the subsequent sample collection, the increase in the number of samples in the validation set will make the model using LDA dimensionality reduction more advanced in terms of computational tasks and recognition time. Compared with PCA and LDA, the SPA algorithm has a relatively poor ability to reduce dimensionality and improve model quality.

The overall effect of KNN and RF among the three machine learning algorithms was better, and the models built had an average RA of up to 80% and an average AUC of up to 0.72, which were significantly better than the SVM model. The best recognition model for DPC appeared in the KNN model as DPC-CWT-LDA-KNN with features number 6, RA = 86.90% and AUC = 0.83; the lowest feature number, the highest recognition accuracy and AUC value made the model own the best recognition performance and good generalization capacity for different white tea production provinces.

Overall, NIRS has great potential to build recognition models for geographically large production areas (different provinces) of white tea. When it comes to building geographically larger production area identification models, the preprocessing algorithms CWT and SNV showed stronger general adaptability, and the combination of the three machine learning algorithms presented significant advantages in identifying white tea origins, leading to a point similar to the results of the study by Zhang et al. [19]. It is suggested that SNV or CWT be combined with other classification algorithms for white tea origin tracing during the subsequent research. The better feature extraction algorithms were PCA and LDA, while the effect of SPA was relatively poor, presumably because LDA and PCA extracted feature vectors, which could represent most of the spectral information; while SPA extracted feature wavenumbers which might not be as comprehensive as feature vectors in terms of representativeness. The overall effect of KNN and RF in the machine learning algorithm was better, and the average RA of the established model could reach 80% and the average AUC could reach 0.72, which was significantly better than the SVM model. To obtain the optimal DPC white tea classification model, the parameters of the DPC-CWT-LDA-KNN model with the best tracking effect on the geographically larger white-tea-producing areas would be optimized subsequently.

#### 3.4.2. Models Evaluation of White Tea Origins Classified by DDFP and AFWT

Table 3 and Table 4 show the evaluation parameters of the same NIRS dataset combined with different pre-processing, feature extraction and machine learning algorithms obtained for identifying geographically narrow range of origin (DDFP) and authenticity of China-Europe Geographical Indication Product (AFWT) models. Since the data sets used were the same, the number of samples in the training set was 291 for all models and the number of samples in the validation set was 98 for all models in Table 3 and Table 4.

The models in Table 3 identify white tea in geographically narrow origin ranges (DDFP, including FD, FA, ZH, SX, JY, and ZR), and the RA of DDFP identification models ranged from 50.00 to 92.86%, and the AUC was in the range of 0.50 to 0.92. The vast majority of DDFP models had RA > 70% and AUC > 0.70, which indicated that the NIR spectral data used were highly correlated with the classification and identification of white tea in a geographically narrow range of origin, and that NIRS combined with machine learning algorithms could achieve fast and nondestructive identification of white tea in a geographically narrow range of origin. The models in Table 4 could make the identification of Fujian white tea as Fuding white tea or not (AFWT, including FD and Non-FD). The RA of AFWT identification models ranged from 51.02 to 97.96%, and the AUC was in the range of 0.50 to 0.98, with the majority of models having RA > 80% and AUC > 0.80, and the models had excellent performance. By comparing the performance difference between AFWT recognition models and DDFP recognition models, it could be seen that when used with the same dataset to reach different recognition goals (differentiated by DDFP or AFWT), the recognition goals with fewer categories were easier to reach, and the RA and AUC values were significantly higher. This may be due to the fact that it was easier to extract the appropriate features when there were fewer recognition categories, improving the model’s quality.

In the established DDFP recognition model, the RA of the KNN, RF and SVM models based on OS were 54.08%, 59.18% and 61.22%, respectively, and the AUC values were 0.62, 0.64 and 0.61, respectively. After OS was preprocessed by CWT, Minmax, MSC and SNV, the RA and AUC of the initially established KNN and RF models were significantly improved, and the model accuracy and generalization ability were improved. In the DDFP recognition model established by SVM algorithm, the quality of the spectral model decreases after MSC preprocessing, and other preprocessing algorithms improve the model’s quality. In the AFWT recognition model, the RA of KNN, RF and SVM models based on OS were 75.51%, 76.53% and 84.69%, respectively, and the AUC were 0.76, 0.77 and 0.85, respectively. After pretreatment with CWT, Minmax, MSC and SNV, the RA and AUC of the initial identification model were significantly improved, and the model accuracy and generalization ability were improved. Like RA and AUC, the preprocessing algorithm performs better in establishing the AFWT recognition model. It is speculated when there were fewer recognition categories, the universality of the preprocessing algorithms would be wider.

After further combination with the feature extraction algorithms, the data dimensions of the DDFP and AFWT recognition models were greatly reduced. In the DDFP recognition models, more than half of the model’s performance was further improved due to the feature extraction algorithms. After a dimensionality reduction using PCA, LDA and SPA algorithms, the number of models whose model quality was further improved was 4.15 and 4, respectively. In the AFWT recognition models, most of the model performance was not further improved by the feature extraction algorithms; after dimensionality reduction using PCA, LDA and SPA algorithms, the number of models whose model quality was further improved was 3.13 and 0, respectively. In general, when establishing DDFP and AFWT recognition models based on the same data, LDA performs best in feature extraction algorithms, and the extracted model has the least number of features and the best model performance. As the number of subsequent samples increases, the increase in the number of validation set samples will make the model using LDA dimensionality reduction more obvious in terms of computing tasks and recognition time.

In the DDFP and AFWT recognition models, the machine learning algorithm RF has the best overall effect, with the highest average RA and AUC values. The best DDFP recognition model appeared in the KNN model, which was DDFP-OS-LDA-KNN with a number of features 5, RA = 92.86%, AUC = 0.92, indicating that the model had good prediction ability for DDFP recognition of white tea; the lowest number of features enables the model to have fewer computing tasks and better computing speed when the number of samples in the subsequent validation set increases. There were three best AFWT recognition models, namely AFWT-OS-LDA-KNN, AFWT-OS-LDA-RF and AFWT-OS-LDA-SVM. Their feature numbers were all 1, RA = 97.96%, and AUC = 0.98. In order to further explore their performance differences, four-fold cross-validation results were introduced to evaluate these three models.

The principle of four-fold cross-validation is to divide the original data set into four subsets equally and make the data of each subset into a validation set, respectively, and the data of the remaining three subsets as the training set, which can get four models performance parameters, and the average of these four models RA is the four-fold cross-validation result. The higher RA of the cross-validation results represents the stronger generalization ability of the model and the better prediction ability for new samples. Table 5 shows the four-fold cross-validation results of AFWT-OS-LDA-KNN, AFWT-OS-LDA-RF and AFWT-OS-LDA-SVM. As shown in the table, the four-fold cross-validation could distinguish small differences in generalization ability among the models when the RA values of the training and validation sets of the three models were the same. AFWT-OS-LDA-KNN had the highest four-fold cross-validation RA of 97.96%, which indicated that KNN was more suitable than the RF and SVM algorithms for the construction of authenticity models with fewer classification categories. To obtain the optimal AFWT identification model, the AFWT-OS-LDA-KNN would be subsequently optimized for the model parameters.

In general, the NIRS dataset combined with different pre-processings, feature extractions and machine learning algorithms was excellent for identifying the geographically narrow range of origin (DDFP) and authenticity of China-Europe Geographical Indication Product (AFWT). SNV performed the best among the preprocessing algorithms and improved the model quality best, with similar findings in the study by Zhang et al. [19]. LDA performs best among the feature extraction algorithms, with the least number of dimensions obtained by dimensionality reduction, which could significantly reduce the model computational task and thus improve the computing speed. Machine learning algorithms with RF in combination with different algorithms present good model results with higher overall average performance parameters; however, the best performing models were found in the KNN model. It is suggested that a reference standard for higher-quality model evaluation parameters can be modeled using the RF algorithm in subsequent studies, and then the KNN algorithm can be used to build a higher-quality model.

#### 3.4.3. Models Optimization

To further improve the performance of the model, the parameters of the three models with the best comprehensive performance in the three types of identification models were optimized, including DPC-CWT-LDA-KNN, DDFP-OS-LDA-KNN and AFWT-OS-LDA-KNN.

**Figure 5:**
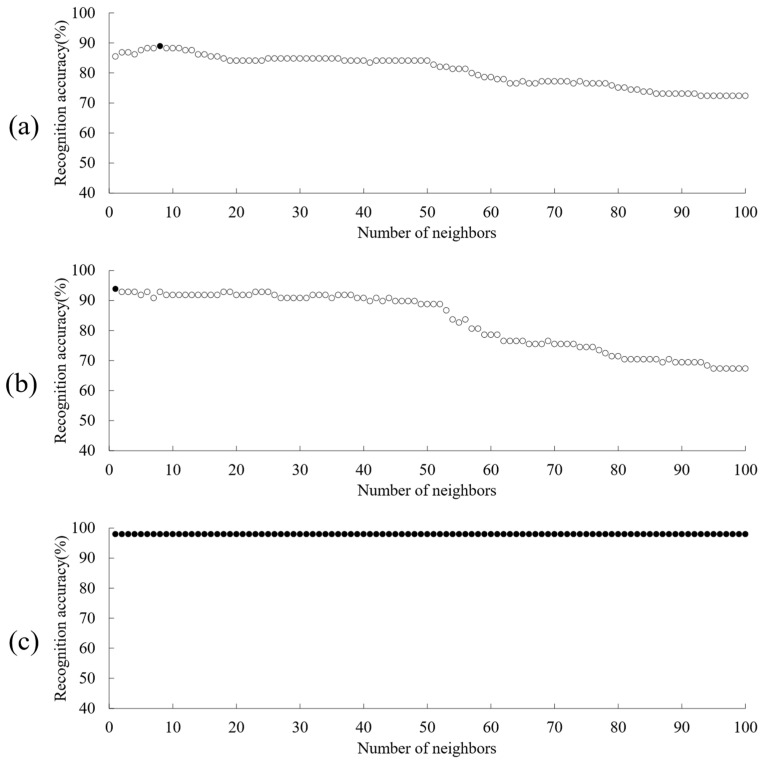
Model performance optimization. (**a**) DPC-CWT-LDA-KNN; (**b**) DDFP-OS-LDA-KNN; (**c**) AFWT-OS-LDA-KNN. (●—Optimal RA).

In the KNN algorithm, the number of neighbors k plays a decisive role in the quality of the model [38]. To further optimized the models, the k-values were defined between 1–100 for model optimization, and the established models were evaluated by the magnitude of RA values as the model performance. Figure 5 shows the curves of RA values for the optimization of parameter k in the above KNN model. The black circle represents the occurrence of the maximum value of RA at that parameter. Thus, the optimal parameter k = 8 when the RA of the DPC-CWT-LDA-KNN validation set reached a maximum value of 88.97% (Figure 5a); the optimal RA of the DDFP-OS-LDA-KNN validation set was 93.88%, when k = 1 (Figure 5b); and the RA of the AFWT-OS-LDA-KNN did not fluctuate due to the change in the value of k (Figure 5c), indicated that the value of k has less influence on the performance of this model, and therefore the model subsequently remains with the initial parameter k = 3.

#### 3.4.4. Performance Analysis of Optimal Models

After the models’ evaluation and optimization, we obtained the best models for identifying white tea DPC, DDFP and AFWT, and the optimal model parameters are shown in Table 6. As shown in the table, the best models for identifying and classifying white tea based on a geographically larger production area (DPC), narrow range of origin (DDFP) and authenticity of China-Europe Geographical Indication Product (AFWT) all had modeling feature numbers less than 10, with the RA all close to or greater than 90% and AUC values all close to or greater than 0.90, which indicated these models possessed excellent prediction and generalization abilities. The excellent quality of the above models demonstrates the ability of NIRS for rapid and nondestructive origin tracing of white tea and provides a reference for other agricultural products in terms of technology and algorithm application in origin traceability.

**Figure 6:**
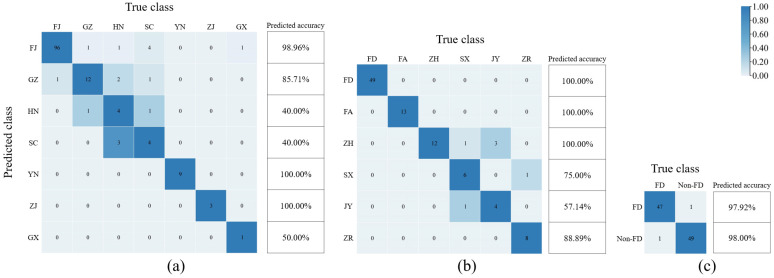
Confusion matrix for the best models. (**a**) DPC-CWT-LDA-KNN; (**b**) DDFP-OS-LDA-KNN; (**c**) AFWT-OS-LDA-KNN.

To further evaluate the ability of the best models to recognize each category, confusion matrices were introduced for in-depth evaluation. The confusion matrix provides a detailed reflection of the performance of the classification model, where the rows represent the true class, and the columns represent the predicted class. The confusion matrix enables the visualization of the number of the correctly classified as well as the categories and number of misclassified categories for each white-tea-producing area. The higher the value on the diagonal of the matrix, the better the prediction ability of the model. The confusion matrix of the best DPC, DDFP and AFWT identification models are shown in Figure 6. From Figure 6a, it could be seen that in distinguishing white tea from different provinces, the predicted accuracy of DPC-CWT-LDA-KNN for YN and ZJ was 100%, and the predicted accuracy for both FJ and GZ was greater than 85.00%; the misclassification occurred mostly in HN and SC samples, and the HN production area was often misclassified as SC production area, and the SC production area was often misclassified as FJ production area. When identifying white tea from different production districts in Fujian Province, the predicted accuracy of DDFP-OS-LDA-KNN for FD, FA and ZH was 100%; misclassification occurred in SX, JY and ZR, and the JY production district was often misclassified as ZH (Figure 6b). As shown in Figure 6c, when performing authenticity identification of Fuding white tea, AFWT-OS-LDA-KNN correctly identified 97.92% and 98.00% of FD and Non-FD, respectively, with excellent prediction ability and good model performance. Overall, the models had excellent correct identification rates for each appellation, and misclassifications occurred mostly among appellations bordering geographic locations. The high similarity of geographic environment, climatic factors and processing processes may be the reason for the frequent misclassification among these appellations.

**Figure 7:**
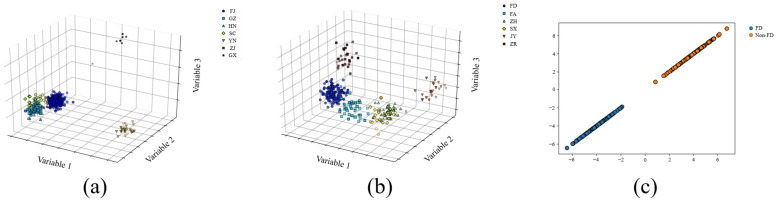
Visualization of the best models for white tea origin traceability. (**a**) DPC-CWT-LDA-KNN; (**b**) DDFP-OS-LDA-KNN; (**c**) AFWT-OS-LDA-KNN.

The optimal model was visualized to reveal the clustering trend of samples from each producing area (Figure 7). In the three-dimensional model diagram of DPC-CWT-LDA-KNN (Figure 7a), the clustering effect of the GX and YN samples was excellent, which could be clearly distinguished from tea in other provinces. The FJ sample’s clustering effect was good, which could be basically separated from other provinces. The spectral characteristics of GZ, HN, SC and ZJ samples were very close to each other in three-dimensional space, the geographical location of these provinces and the similarity of the climatic conditions and tea processing technology may be the reasons for the clustering effect’s good performance. By observing the three-dimensional model diagram of DDFP-OS-LDA-KNN (Figure 7b), it could be found that samples from different producing areas could basically be clustered separately in three-dimensional space, which could easily distinguish them. ZH and SX samples were close, which may be due to their similar geographical locations and similar processing technology. By observing the visualization of the AFWT-OS-LDA-KNN model (Figure 7c), it could be found that the clustering of FD and Non-FD samples were very effective, which may be the reason for the excellent effect of the model. In general, after visualization, the distribution of spectral characteristics of white tea samples in some producing areas was very close in three-dimensional space, which may be related to the small number of samples collected in these producing areas and the lack of obvious spectral characteristics. In addition, it may also be related to the similarity of the white tea quality caused by geographical location, climatic factors and similar processing technology. To solve these problems, we will strengthen the spectral characteristics of the production area by increasing the number of samples year by year, so as to further improve the performance of the model.

## 4. Conclusions

This study proved the feasibility of using NIRS data to verify the origin of white tea simply and quickly. Combining different spectra data preprocessing methods (CWT, Minmax, MSC and SNV) with different feature extraction algorithms (PCA, LDA and SPA), 180 white tea origin traceability models were established based on KNN, RF and SVM algorithms. The modeling results show that the SNV effect was the most excellent among the preprocessing algorithms, and the performance of the model was improved best without combining other algorithms. LDA has the greatest advantages in different feature extraction algorithms, and the number of features obtained by dimensionality reduction was the least. RF has the strongest general adaptability in machine learning algorithms, but the best model quality generally appears in KNN models. DPC-CWT-LDA-KNN, DDFP-OS-LDA-KNN and AFWT-OS-LDA-KNN were proved to be the optimal models for identifying white tea origins classified by DPC, DDFP and AFWT. The RA of the optimal models was close to or greater than 90%, and their AUC value was close to or greater than 0.90, these models had excellent predictive ability and good generalization ability. Overall, this study demonstrates the possibility of achieving white tea origin traceability based on NIRS, representing a step forward in the method selection for origin traceability and quality control of white tea. Based on the above research results, in order to solve the problems of unbalanced model samples and the close distance of model clusters, we will increase the number of samples year by year in the future, enrich the white tea NIRS data set to optimize model performance and develop portable white tea origin traceability devices on this basis. In addition, we will also try to build an online white tea origin identification platform using internet technology to carry out remote white tea origin traceability.

## Figures and Tables

**Table 1:** PCA eigenvalues and cumulative contribution.

NIRS Data Matrix	Number of Principal Components	OS		CWT		Minmax		SNV		MSC	
		Eigenvalue	Cumulative Contribution	Eigenvalue	Cumulative Contribution	Eigenvalue	Cumulative Contribution	Eigenvalue	Cumulative Contribution	Eigenvalue	Cumulative Contribution
White tea origins classified by DPC	1	806.91	97.10%	470.66	56.64%	573.28	69.07%	614.11	73.90%	614.35	73.93%
	2	15.94	99.02%	161.16	76.03%	187.72	91.69%	146.26	91.50%	145.78	91.47%
	3	5.48	99.68%	117.55	90.18%	49.09	97.60%	44.73	96.88%	44.35	96.81%
	4	1.72	99.89%	38.38	94.80%	10.90	98.91%	10.87	98.19%	10.85	98.11%
	5	0.47	99.94%	21.34	97.36%	3.77	99.37%	8.64	99.23%	9.23	99.23%
	6	0.21	99.97%	6.00	98.09%	1.51	99.55%	2.20	99.50%	2.38	99.51%
	7	0.16	99.99%	4.39	98.61%	1.13	99.69%	1.70	99.70%	1.63	99.71%
	8	0.04	99.99%	3.53	99.04%	0.87	99.79%	0.66	99.78%	0.67	99.79%
	9	0.03	99.99%	2.80	99.38%	0.55	99.86%	0.53	99.84%	0.61	99.86%
	10	0.02	100.00%	1.48	99.55%	0.36	99.90%	0.39	99.89%	0.35	99.90%
	11	0.01	100.00%	1.27	99.71%	0.23	99.93%	0.21	99.92%	0.17	99.92%
	12	0.01	100.00%	0.68	99.79%	0.14	99.94%	0.15	99.93%	0.15	99.94%
	13	0.00	100.00%	0.48	99.85%	0.10	99.96%	0.13	99.95%	0.12	99.96%
	14	0.00	100.00%	0.35	99.89%	0.08	99.97%	0.10	99.96%	0.08	99.97%
	15	0.00	100.00%	0.26	99.92%	0.05	99.97%	0.07	99.97%	0.07	99.97%
White tea origins classified by DDFP or AFWT	1	808.20	97.26%	436.20	52.49%	563.59	67.90%	625.87	75.32%	626.54	75.40%
	2	13.37	98.87%	209.90	77.75%	204.63	92.56%	141.17	92.30%	140.95	92.36%
	3	7.53	99.77%	92.95	88.94%	41.72	97.58%	34.41	96.44%	34.28	96.48%
	4	1.04	99.90%	58.57	95.98%	10.56	98.86%	13.20	98.03%	12.71	98.01%
	5	0.53	99.96%	11.19	97.33%	4.11	99.35%	10.58	99.31%	10.93	99.33%
	6	0.16	99.98%	6.75	98.14%	1.98	99.59%	1.88	99.53%	1.75	99.54%
	7	0.07	99.99%	5.16	98.76%	0.92	99.70%	1.38	99.70%	1.39	99.70%
	8	0.05	99.99%	3.39	99.17%	0.72	99.78%	0.74	99.79%	0.84	99.81%
	9	0.02	100.00%	2.05	99.42%	0.58	99.85%	0.55	99.85%	0.56	99.87%
	10	0.01	100.00%	1.36	99.58%	0.34	99.89%	0.30	99.89%	0.26	99.90%
	11	0.01	100.00%	0.99	99.70%	0.21	99.92%	0.20	99.91%	0.20	99.93%
	12	0.00	100.00%	0.66	99.78%	0.16	99.94%	0.19	99.94%	0.14	99.95%
	13	0.00	100.00%	0.57	99.85%	0.12	99.95%	0.13	99.95%	0.13	99.96%
	14	0.00	100.00%	0.31	99.89%	0.08	99.96%	0.10	99.97%	0.07	99.97%
	15	0.00	100.00%	0.26	99.92%	0.06	99.97%	0.07	99.97%	0.05	99.98%

**Table 2:** Performance evaluation of traceability models of white tea classified by DPC.

Classification Model	Number of Samples Trained	Number of Samples Predicted	Number of Characteristics	RA (%)	AUC	Average RA (%)	Average AUC
DPC-OS-KNN	434	145	831	73.10	0.62	80.10	0.72
DPC-CWT-KNN			831	76.55	0.67		
DPC-Minmax-KNN			831	81.38	0.70		
DPC-MSC-KNN			831	81.38	0.71		
DPC-SNV-KNN			831	81.38	0.71		
DPC-OS-PCA-KNN			4	73.10	0.62		
DPC-CWT-PCA-KNN			11	76.55	0.67		
DPC-Minmax-PCA-KNN			7	82.07	0.71		
DPC-MSC-PCA-KNN			7	81.38	0.71		
DPC-SNV-PCA-KNN			7	81.38	0.71		
DPC-OS-LDA-KNN			6	84.14	0.82		
DPC-CWT-LDA-KNN			6	86.90	0.83		
DPC-Minmax-LDA-KNN			6	84.14	0.83		
DPC-MSC-LDA-KNN			6	85.52	0.81		
DPC-SNV-LDA-KNN			6	85.52	0.83		
DPC-OS-SPA-KNN			15	73.10	0.62		
DPC-CWT-SPA-KNN			13	75.86	0.64		
DPC-Minmax-SPA-KNN			13	77.24	0.68		
DPC-MSC-SPA-KNN			15	80.69	0.74		
DPC-SNV-SPA-KNN			11	80.69	0.71		
DPC-OS-RF	434	145	831	75.17	0.65	80.00	0.73
DPC-CWT-RF			831	83.45	0.71		
DPC-Minmax-RF			831	80.00	0.68		
DPC-MSC-RF			831	83.45	0.78		
DPC-SNV-RF			831	82.76	0.77		
DPC-OS-PCA-RF			4	79.31	0.70		
DPC-CWT-PCA-RF			11	84.83	0.76		
DPC-Minmax-PCA-RF			7	86.90	0.82		
DPC-MSC-PCA-RF			7	83.45	0.80		
DPC-SNV-PCA-RF			7	84.83	0.81		
DPC-OS-LDA-RF			6	73.10	0.67		
DPC-CWT-LDA-RF			6	81.38	0.74		
DPC-Minmax-LDA-RF			6	68.97	0.70		
DPC-MSC-LDA-RF			6	77.24	0.70		
DPC-SNV-LDA-RF			6	71.72	0.68		
DPC-OS-SPA-RF			15	74.48	0.63		
DPC-CWT-SPA-RF			13	82.07	0.77		
DPC-Minmax-SPA-RF			13	80.00	0.70		
DPC-MSC-SPA-RF			15	82.07	0.74		
DPC-SNV-SPA-RF			11	84.83	0.76		
DPC-OS-SVM	434	145	831	75.86	0.63	74.38	0.61
DPC-CWT-SVM			831	77.24	0.64		
DPC-Minmax-SVM			831	75.17	0.64		
DPC-MSC-SVM			831	73.10	0.59		
DPC-SNV-SVM			831	82.07	0.74		
DPC-OS-PCA-SVM			4	75.86	0.63		
DPC-CWT-PCA-SVM			11	77.24	0.64		
DPC-Minmax-PCA-SVM			7	75.17	0.64		
DPC-MSC-PCA-SVM			7	73.10	0.59		
DPC-SNV-PCA-SVM			7	82.07	0.73		
DPC-OS-LDA-SVM			6	71.72	0.57		
DPC-CWT-LDA-SVM			6	77.93	0.67		
DPC-Minmax-LDA-SVM			6	73.10	0.58		
DPC-MSC-LDA-SVM			6	75.17	0.63		
DPC-SNV-LDA-SVM			6	72.41	0.61		
DPC-OS-SPA-SVM			15	70.34	0.56		
DPC-CWT-SPA-SVM			13	68.97	0.54		
DPC-Minmax-SPA-SVM			13	71.72	0.58		
DPC-MSC-SPA-SVM			15	66.90	0.50		
DPC-SNV-SPA-SVM			11	72.41	0.58		

**Table 3:** Performance evaluation of traceability models of white tea classified by DDFP.

Classification Model	Number of Samples Trained	Number of Samples Predicted	Number of Characteristics	RA (%)	AUC	Average RA (%)	Average AUC
DDFP-OS-KNN	291	98	831	54.08	0.62	71.48	0.74
DDFP-CWT-KNN			831	65.31	0.68		
DDFP-Minmax-KNN			831	70.41	0.73		
DDFP-MSC-KNN			831	66.33	0.69		
DDFP-SNV-KNN			831	66.33	0.69		
DDFP-OS-PCA-KNN			4	53.06	0.61		
DDFP-CWT-PCA-KNN			10	65.31	0.68		
DDFP-Minmax-PCA-KNN			6	69.39	0.73		
DDFP-MSC-PCA-KNN			7	65.31	0.68		
DDFP-SNV-PCA-KNN			7	65.31	0.68		
DDFP-OS-LDA-KNN			5	92.86	0.92		
DDFP-CWT-LDA-KNN			5	82.65	0.85		
DDFP-Minmax-LDA-KNN			5	88.78	0.89		
DDFP-MSC-LDA-KNN			5	90.82	0.91		
DDFP-SNV-LDA-KNN			5	90.82	0.91		
DDFP-OS-SPA-KNN			13	54.08	0.62		
DDFP-CWT-SPA-KNN			19	75.51	0.77		
DDFP-Minmax-SPA-KNN			12	67.35	0.70		
DDFP-MSC-SPA-KNN			14	74.49	0.75		
DDFP-SNV-SPA-KNN			13	71.43	0.74		
DDFP-OS-RF	291	98	831	59.18	0.64	75.56	0.78
DDFP-CWT-RF			831	72.45	0.75		
DDFP-Minmax-RF			831	75.51	0.80		
DDFP-MSC-RF			831	76.53	0.80		
DDFP-SNV-RF			831	76.53	0.80		
DDFP-OS-PCA-RF			4	66.33	0.68		
DDFP-CWT-PCA-RF			10	75.51	0.78		
DDFP-Minmax-PCA-RF			6	77.55	0.79		
DDFP-MSC-PCA-RF			7	76.53	0.79		
DDFP-SNV-PCA-RF			7	76.53	0.80		
DDFP-OS-LDA-RF			5	85.71	0.89		
DDFP-CWT-LDA-RF			5	80.61	0.81		
DDFP-Minmax-LDA-RF			5	85.71	0.86		
DDFP-MSC-LDA-RF			5	86.73	0.88		
DDFP-SNV-LDA-RF			5	81.63	0.86		
DDFP-OS-SPA-RF			13	54.08	0.60		
DDFP-CWT-SPA-RF			19	76.53	0.80		
DDFP-Minmax-SPA-RF			12	74.49	0.77		
DDFP-MSC-SPA-RF			14	77.55	0.79		
DDFP-SNV-SPA-RF			13	75.51	0.77		
DDFP-OS-SVM	291	98	831	61.22	0.61	70.34	0.68
DDFP-CWT-SVM			831	69.39	0.69		
DDFP-Minmax-SVM			831	73.47	0.77		
DDFP-MSC-SVM			831	58.16	0.58		
DDFP-SNV-SVM			831	76.53	0.79		
DDFP-OS-PCA-SVM			4	75.86	0.63		
DDFP-CWT-PCA-SVM			10	77.24	0.64		
DDFP-Minmax-PCA-SVM			6	75.17	0.64		
DDFP-MSC-PCA-SVM			7	73.10	0.59		
DDFP-SNV-PCA-SVM			7	82.07	0.73		
DDFP-OS-LDA-SVM			5	89.80	0.90		
DDFP-CWT-LDA-SVM			5	80.61	0.82		
DDFP-Minmax-LDA-SVM			5	81.63	0.83		
DDFP-MSC-LDA-SVM			5	86.73	0.86		
DDFP-SNV-LDA-SVM			5	86.73	0.86		
DDFP-OS-SPA-SVM			13	50.00	0.50		
DDFP-CWT-SPA-SVM			19	50.00	0.50		
DDFP-Minmax-SPA-SVM			12	54.08	0.53		
DDFP-MSC-SPA-SVM			14	50.00	0.50		
DDFP-SNV-SPA-SVM			13	55.10	0.55		

**Table 4:** Performance Evaluation of Traceability Models of White Tea Classified by AFWT.

Classification Model	Number of Samples Trained	Number of Samples Predicted	Number of Characteristics	RA (%)	AUC	Average RA (%)	Average AUC
AFWT-OS-KNN	291	98	831	75.51	0.76	85.10	0.83
AFWT-CWT-KNN			831	77.55	0.78		
AFWT-Minmax-KNN			831	88.78	0.89		
AFWT-MSC-KNN			831	89.80	0.90		
AFWT-SNV-KNN			831	89.80	0.90		
AFWT-OS-PCA-KNN			4	73.47	0.74		
AFWT-CWT-PCA-KNN			10	77.55	0.78		
AFWT-Minmax-PCA-KNN			6	88.78	0.89		
AFWT-MSC-PCA-KNN			7	88.78	0.89		
AFWT-SNV-PCA-KNN			7	88.78	0.89		
AFWT-OS-LDA-KNN			1	97.96	0.98		
AFWT-CWT-LDA-KNN			1	94.90	0.95		
AFWT-Minmax-LDA-KNN			1	92.86	0.93		
AFWT-MSC-LDA-KNN			1	94.90	0.95		
AFWT-SNV-LDA-KNN			1	94.90	0.95		
AFWT-OS-SPA-KNN			11	73.10	0.62		
AFWT-CWT-SPA-KNN			13	75.86	0.64		
AFWT-Minmax-SPA-KNN			10	77.24	0.68		
AFWT-MSC-SPA-KNN			12	80.69	0.74		
AFWT-SNV-SPA-KNN			13	80.69	0.71		
AFWT-OS-RF	291	98	831	76.53	0.77	89.21	0.87
AFWT-CWT-RF			831	90.82	0.91		
AFWT-Minmax-RF			831	91.84	0.92		
AFWT-MSC-RF			831	93.88	0.94		
AFWT-SNV-RF			831	95.92	0.96		
AFWT-OS-PCA-RF			4	82.65	0.83		
AFWT-CWT-PCA-RF			10	92.86	0.93		
AFWT-Minmax-PCA-RF			6	91.84	0.92		
AFWT-MSC-PCA-RF			7	94.90	0.95		
AFWT-SNV-PCA-RF			7	93.88	0.94		
AFWT-OS-LDA-RF			1	97.96	0.98		
AFWT-CWT-LDA-RF			1	94.90	0.95		
AFWT-Minmax-LDA-RF			1	92.86	0.93		
AFWT-MSC-LDA-RF			1	94.90	0.95		
AFWT-SNV-LDA-RF			1	94.90	0.95		
AFWT-OS-SPA-RF			11	74.49	0.63		
AFWT-CWT-SPA-RF			13	82.07	0.77		
AFWT-Minmax-SPA-RF			10	80.00	0.70		
AFWT-MSC-SPA-RF			12	82.07	0.74		
AFWT-SNV-SPA-RF			13	84.83	0.76		
AFWT-OS-SVM	291	98	831	84.69	0.85	82.85	0.80
AFWT-CWT-SVM			831	88.78	0.89		
AFWT-Minmax-SVM			831	90.82	0.91		
AFWT-MSC-SVM			831	89.80	0.90		
AFWT-SNV-SVM			831	94.90	0.95		
AFWT-OS-PCA-SVM			4	75.86	0.63		
AFWT-CWT-PCA-SVM			10	77.24	0.64		
AFWT-Minmax-PCA-SVM			6	75.17	0.64		
AFWT-MSC-PCA-SVM			7	73.10	0.59		
AFWT-SNV-PCA-SVM			7	82.07	0.73		
AFWT-OS-LDA-SVM			1	97.96	0.98		
AFWT-CWT-LDA-SVM			1	94.90	0.95		
AFWT-Minmax-LDA-SVM			1	92.86	0.93		
AFWT-MSC-LDA-SVM			1	94.90	0.95		
AFWT-SNV-LDA-SVM			1	94.90	0.95		
AFWT-OS-SPA-SVM			11	66.33	0.66		
AFWT-CWT-SPA-SVM			13	65.31	0.66		
AFWT-Minmax-SPA-SVM			10	81.63	0.82		
AFWT-MSC-SPA-SVM			12	51.02	0.50		
AFWT-SNV-SPA-SVM			13	84.70	0.85		

**Table 5:** Four times cross-validation results of the optimal model for authenticity identification of Fuding white tea.

Classification Model	RA (%)		Four Times Cross-Validation RA (%)
	Training Set	Validation Set	
AFWT-OS-LDA-KNN	100.00	97.96	97.96
AFWT-OS-LDA-RF	100.00	97.96	95.87
AFWT-OS-LDA-SVM	100.00	97.96	96.96

**Table 6:** Optimal model performance parameters.

Optimal Models	Parameters	Number of Characteristics	RA (%)	AUC
DPC-CWT-LDA-KNN	k = 8	6	88.97	0.85
DDFP-OS-LDA-KNN	k = 1	5	93.88	0.93
AFWT-OS-LDA-KNN	k = 3	1	97.96	0.98

## Data Availability

The data presented in this study are available on request from the corresponding author.
